# Transformation and Precipitation Reactions by Metal Active Gas Pulsed Welded Joints from X2CrNiMoN22-5-3 Duplex Stainless Steels

**DOI:** 10.3390/ma9070606

**Published:** 2016-07-21

**Authors:** Ion-Dragos Utu, Ion Mitelea, Sorin Dumitru Urlan, Corneliu Marius Crăciunescu

**Affiliations:** Department of Materials and Manufacturing Engineering, University Politehnica Timisoara, Pta Victoriei, No. 2, Timisoara 300006, Romania; dragos.utu@upt.ro (I.-D.U.); sorinurlan@yahoo.com (S.D.U.); craciunescucm@yahoo.com (C.M.C.)

**Keywords:** Duplex stainless steel, MAG pulsed welding, structure

## Abstract

The high alloying degree of Duplex stainless steels makes them susceptible to the formation of intermetallic phases during their exposure to high temperatures. Precipitation of these phases can lead to a decreasing of the corrosion resistance and sometimes of the toughness. Starting from the advantages of the synergic Metal Active Gas (MAG) pulsed welding process, this paper analyses the structure formation particularities of homogeneous welded joints from Duplex stainless steel. The effect of linear welding energy on the structure morphology of the welded joints was revealed by macro- and micrographic examinations, X-ray energy dispersion analyses, measurements of ferrite proportion and X-ray diffraction analysis. The results obtained showed that the transformation of ferrite into austenite is associated with the chromium, nickel, molybdenum and nitrogen distribution between these two phases and their redistribution degree is closely linked to the overall heat cycle of the welding process. The adequate control of the energy inserted in the welded components provides an optimal balance between the two microstructural constituents (Austenite and Ferrite) and avoids the formation of undesirable intermetallic phases.

## 1. Introduction

Duplex stainless steel is a two-phase material with an equilibrium microstructure consisting of approximately 50% ferrite (F) and 50% austenite (A). They combine the attractive properties of austenitic and ferritic stainless steels, such as high mechanical strength characteristics, high stability to stress-cracking corrosion and pitting, and crevice corrosion resistance [1,2,3,4]. For these reasons, they are extensively used for mobile offshore plants for oil and gas drilling, desalination of seawater factories, chemical industry installations, food industry equipment, etc. [5,6,7].

The main alloying elements in the chemical composition of these steels are Cr, Ni, Mo and N. Chromium and molybdenum lead to the ferrite formation, stabilizing the body-centred cubic lattice (BCC), while Ni and N have a γ-gene character, stabilizing the face-centred cubic crystal (FCC) austenite lattice. The best exploitation properties are obtained when the two phases proportion is about 50/50 (fifty/fifty) and when the phases like sigma (σ), chi (χ), secondary austenite (γ_2_), carbides and nitrides of chromium, which affects the corrosion resistance and toughness, are not present in the structure [8,9,10,11].

Most of the industrial constructions made of Duplex stainless steels use welding techniques [2,4,6]. The recommended welding processes are: Coated electrode manual welding, TIG (Tungsten Inert Gas) and MIG (Metal Inert Gas)/MAG (Metal Active Gas) welding, flux-cored arc welding, submerged and plasma arc welding. Although it is considered that they have an acceptable welding behaviour, the material melting and solidification during welding processes degrades the favourable microstructure of the base material [12,13].

Casalino et al. [14] showed the feasibility of the welding of the selective laser molten stainless steel by fibre laser-arc hybrid in terms of effectiveness and efficiency.

Previous research has demonstrated that plasma or TIG welding without filler material must be avoided [15].

The TIG joining method is recommended only for one side welding of the root layer; for example, in pipes where manual welding with coated electrodes will be used for filling layers [16]. Butt welded joints will usually be obtained by coated electrodes manual welding and submerged or cored wire welding [17,18].

In order to reshuffle the casted products, manual welding with coated electrodes is typically used [1,8]. Hilkes et al. [19] showed that welding by fusion leads to formation of a coarse ferrite microstructure and austenite separated inter- and intra-granularboth in the weld and in the heat affected zone (HAZ). The volume of ferrite fraction is greater than that of austenite in both parts of the welded joint. Sato et al. [18] have shown that higher ferrite contents and a coarse grain size cause a decrease of the resistance corrosion and mechanical properties. If the linear welding energy is too low, the proportion of ferrite formed in the weld is high (over 70%) and precipitation of chromium nitrides is intense. Instead, a higher linear welding energy and/or an extended exposure at temperatures of 600–900 °C can cause precipitation of intermetallic sigma (σ) or chi (χ) phases [9]. Therefore, the welding specifications must be designed in such a way as to obtain the phases proportions (ratio F/A) close to 1:1 and to avoid the intense precipitation of intermetallic phases, controlling and limiting the heat input at certain values [5,13]. Świerczyński and others [4] concluded that with these steels, the selection of the proper heat input cannot be made from only literature data, which are often divergent. Each case should be considered individually and it is good practice to develop a separate welding technology [17].

For technological and economic reasons, one of the welding processes selected to be put into practice in this paper is synergic pulsed metal active gas (MAG). It has the great advantage that by the periodic changes of the welding current the routed transfer of the droplet through the metal arc takes place. At the same time, it provides the energy control introduced in the components, at lower values compared to spray welding (which is often compared), having a positive effect on reducing stresses and strains in the welding.

## 2. Experimental Procedure

The chemical composition of the steel samples used in this work is detailed in Table 1.

For welding, an inverter power LUC 500 Aristo (Esab Welding Equipment AB, Laxa, Sweden) built in modular system, programmable, with Siemens microprocessor and possibility of PC connection was used.

It is a synergistic welding source which allows the automatic adjustment and control of the heat power by changing the wire electrode feed speed, after the input data had been previously set up, namely: Transfer mode, filler material, shielding gas protection and wire diameter The welding source maintains the pulse current and pulse time constant for given input conditions, modifying together with the wire feed speed only the pulse frequency andthe base current, in order to provide the synergic transfer at different arc powers (electrode wire feed speeds).

The synergic welding programwas established based on the following initial conditions:

- defining of the welded joint: homogeneous;
- *base metal*: Duplex stainless steel sheet: s = 12 mm;
- type of the welded joint: butt penetrated joint;
- weld thickness: 12 mm;
- welding position: horizontal PA;
- welding technique: pulsed MAG;
- filler material: E 2209-16 wire (according to AWS A5.4); it has a Cr, Mo and N content similar to the base metal, but the more Ni (8%–10%);
- diameter wire electrode: d_s_ = 1.2 mm;
- shielding gas: Cronigon 2 (97.5% Ar + 2.5% CO_2_), supplied by Linde Gas;
- flow gas: 18 L/min;
- welding direction: to the left;
- inclination of the electrode wire: 85°.

**Figure 1:**
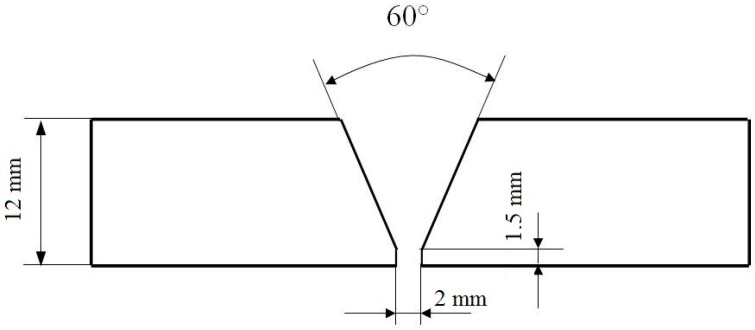
The shape and dimensions of welding joint.

**Figure 2:**
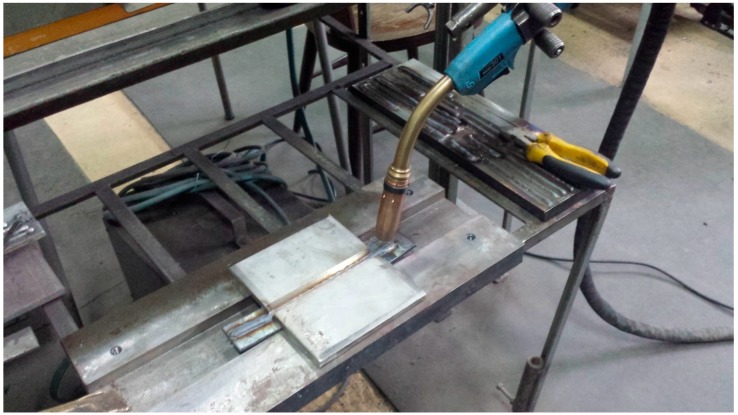
Components and welding torch.

The welding process was done in horizontal position, ISO 6947/2011 [20] position. The preparation of the welding joint with the components and welding gun positioning is presented in Figure 1 and Figure 2, respectively. A butt penetrated weld with access from one side was realized.

It is mentioned that the contact nozzle was positioned within the gas nozzle at 2–3 mm, so that a free end length of 18–19 mm resulted. In these circumstances, several sets of welded joints with three linear energy values, E_l_: 10 kJ/cm, 15 kJ/cm, and 20.7 kJ/cm, were executed.

The linear energy is defined as heat quantity inserted in the material by obtaining of a weld with unitary length [21]. It is calculated with the expression:

Linear energy

E_l_ = (U_arc_ × I_s_)/(100 × v_s_) (1)

where U_arc_ is arc voltage (V); I_s_ is welding current [A]; and v_s_ is welding speed (cm/s).

With each of these, the root layer was realized with a lower linear energy, of 6.9 kJ/cm, in order to prevent the root penetration and leakage of the molten metal. The temperature between the weld passes was limited to 150 °C. To change the linear energy, E_1_, by filling the welded joint, the welding speed or welding current was modified [22].

The structural analyses regarding the welded joints quality, obtained under these conditions, were performed using conventional metallographic techniques. The nature of the present phases was identified by X-ray diffraction, and their chemical composition and microstructure was investigated by TESCAN Vega 3 LM scanning electron microscope (SEM) (TESCAN Brno, Brno, Czech Republic‎), equipped with a Bruker Quantax 200 Energy Dispersive X-ray Spectroscopy (EDX) system with a Peltier-cooled XFlash 410M silicon drift detector (Bruker, Billerica, MA, USA).

## 3. Results and Discussion

### 3.1. Macrographic Aspect of the Welded Joints

In order to examine the structure and to highlight the heterogeneities occurred in welded joints, metallographic samples with transversal faces (perpendicular to the weld longitudinal axis) were collected and prepared, in accordance with the known standard techniques.

**Figure 3:**
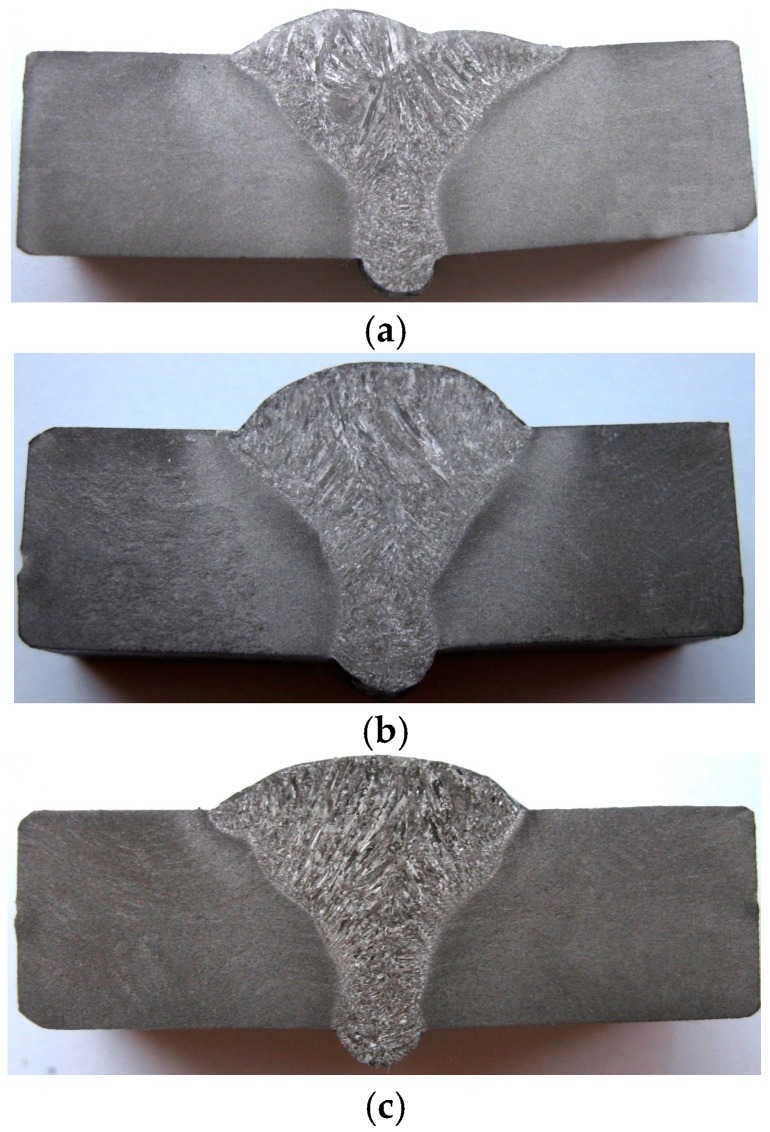
Macroscopic images of the welded joints cross section: (**a**) E_l_ welded joint = 6.9 kJ/cm, E_l_ filling layers = 10 kJ/cm; (**b**) E_l_ welded joint = 6.9 kJ/cm, E_l_ filling layers = 15 kJ/cm; and (**c**) E_l_ welded joint = 6.9 kJ/cm, E_l_ filling layers = 20.7 kJ/cm. Chemical reactive: ferric chloride 10 cm^3^; hydrochloric acid 30 cm^3^; and ethylic alcohol 120 cm^3^.

The macrographic aspect (cross section) of the realized welded joints with constant linear energy for the root layer and variable energy for the filling layers is shown in Figure 3.

Although the welding process provides the material continuity, the welded joint zone does not reveal a homogeneous structure. As a result of high temperatures heating, the melting of the filler material and a portion of the base material appeared, and by subsequent cooling a series of structural modifications were produced. Thus, in the root layer, which was obtained at a constant value of the linear energy, a relatively fine structure is shown, determined on the one hand by the high value of the sub-cooling degree (critical radius of the crystallisation germ is small, and their number is large), and on the other hand by the annealing effect, which occurs during the filling layers deposition.

It can be also notice a slight increase of the heat affected zone (HAZ) width in adjacent portion of the welded joint determined by the multiple thermal cycles overlapping. At the first set of samples, although the linear energy was lower (10 kJ/cm) and the welding speed was higher (30 cm/min.), two weld passes were necessary in order to achieve the final layer deposition. This justifies the slightly different weld profile compared to the other sets of samples and also the larger sizes of the dendritic crystals oriented in the heat evacuation direction (Figure 3a compared to Figure 3b,c).

The linear energy variation within the range between 10 and 20.7 kJ/cm does not cause macroscopic defects such as pores, cracks or slag inclusions in the welded joints.

### 3.2. Microstructure of the Weld Seam and Joint Interface

**Figure 4:**
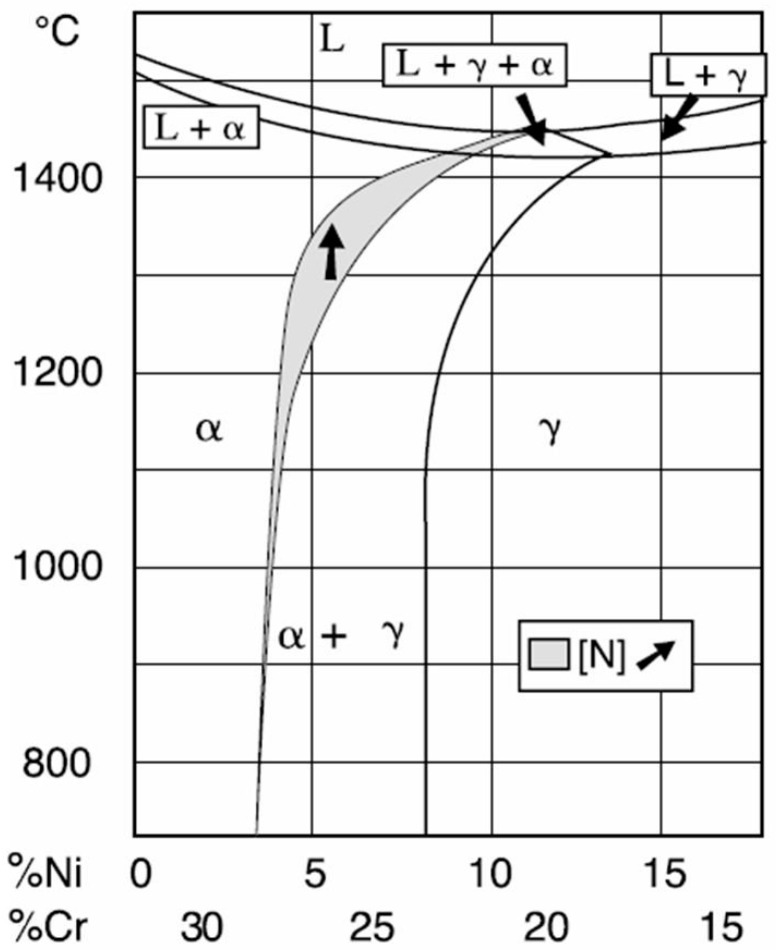
The phases pseudo binary Fe-Cr-Ni diagram for 68% Fe [23,24].

According to the phases’ pseudo binary Fe-Cr-Ni diagram for a 68% Fe concentration, shown in Figure 4, the solidification microstructure of the molten metal bath is almost completely ferritic. By further cooling, the austenite formation on the ferrite grain boundaries is initiated. The amount of austenite depends mainly on the chemical composition and cooling rate.

It can be seen in Figure 4 that even restricted limits modifications of Ni and Cr concentrations significantly influence the proportions of the two phases, ferrite and austenite.

Moreover, depending on the thermal history of steel formation and processing, other microstructural constituents can be formed, such as carbides, nitrides, σ phase and other intermetallic phases [8].

By welding, the cooling rate is relatively high and, subsequently, there is a short time to form austenite. Therefore, the selected filler material has a higher Ni content (element that stabilizes the austenite phase), as compared to the base metal. A similar effect can be obtained by adding N, which has a great importance in reforming austenite.

**Figure 5:**
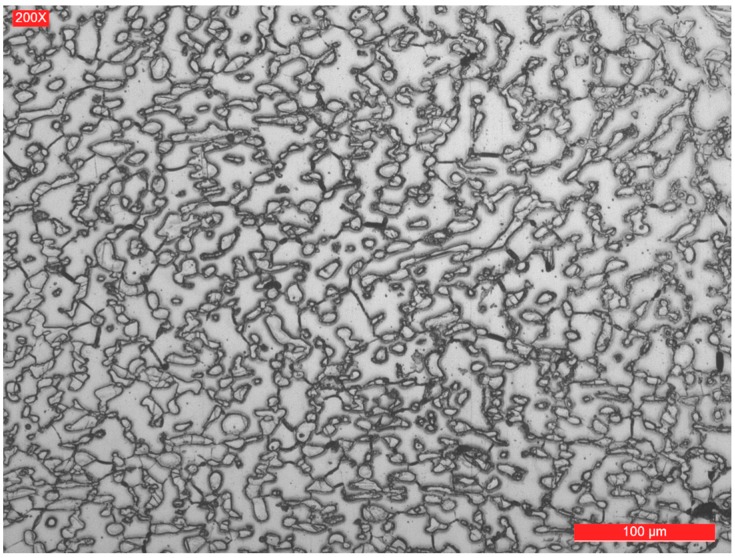
Microstructure of the base metal.

**Figure 6:**
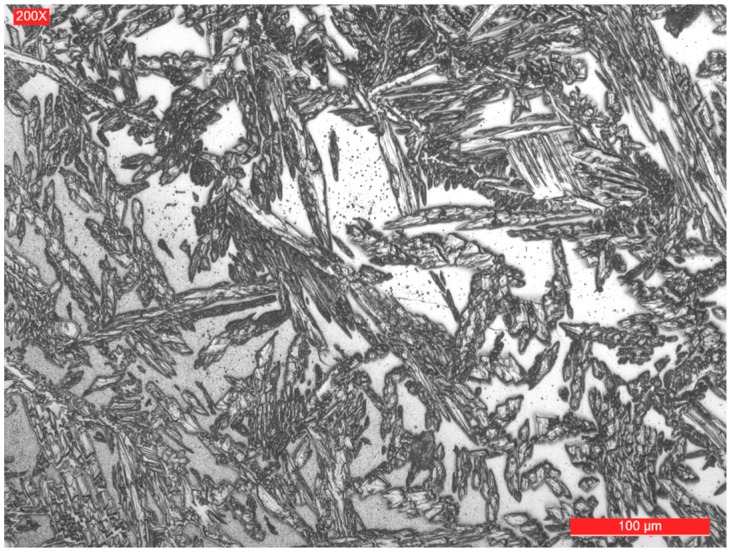
Microstructure of the root layer.

**Figure 7:**
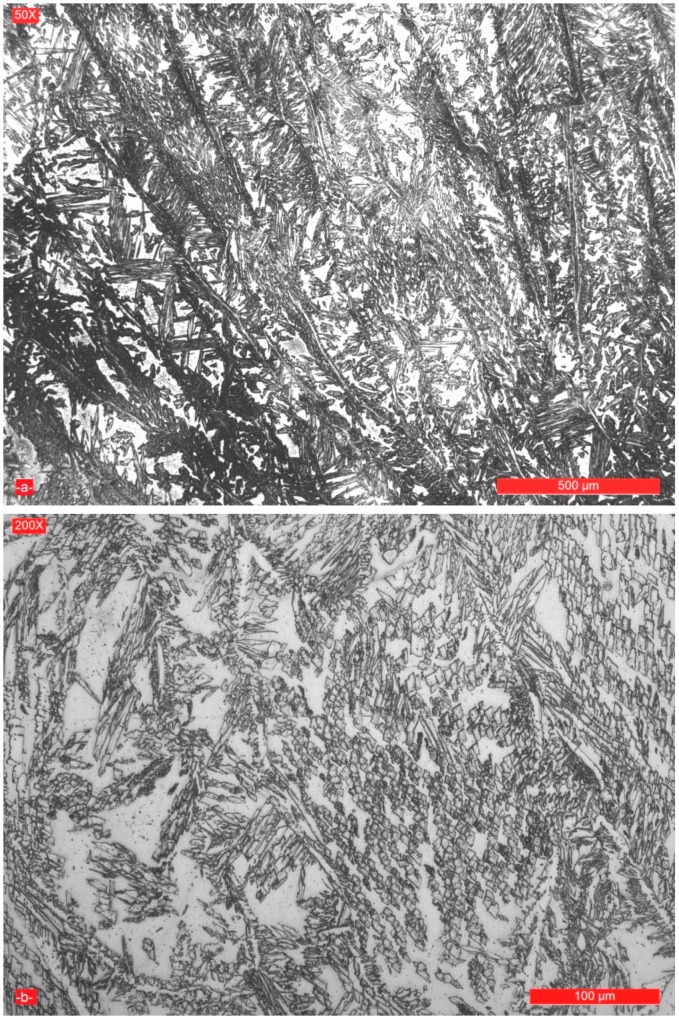
Microstructure of the last deposited layer for achieving the welded joint with a linear energy E_l_ = 15 kJ/cm: (**a**) magnification 50×; and (**b**) magnification 200×.

**Figure 8:**
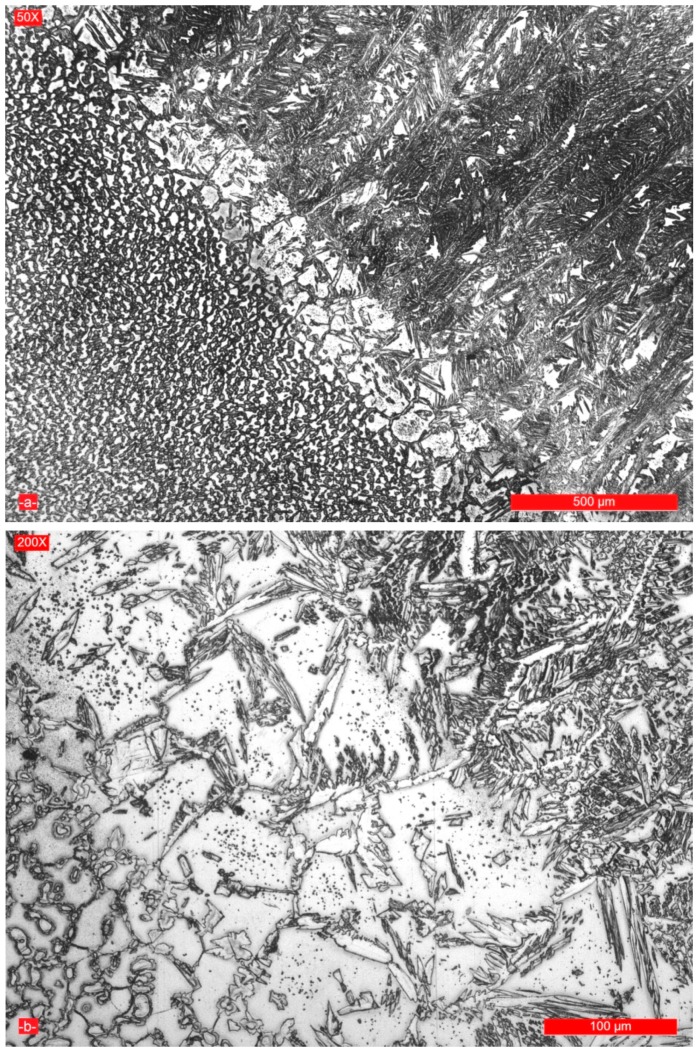
Microstructure of the weld-HAZ interface for achieving the welded joint with a linear energy. E_l_ = 15 kJ/cm: (**a**) magnification 50×; and (**b**) magnification 200×.

The representative microstructures of the welded joint areas are shown in Figure 5, Figure 6, Figure 7 and Figure 8. It can be noticed that if the base metal (Figure 5) has a structure consisting of approximately 51% ferrite and 49% austenite (determined by quantitative metallography), the deposited metal has a structure with dendritic orientation (Figure 6), themorphology of which depends on the welding thermal cycle particularities. Thus, in the welded joint, the thermal cycle is the most severe, the cooling rate is quite high and the formation time of austenite from ferrite is lower. As a result, the quantitative metallographic analysis showed that the proportion of the two phases is 55%–57% ferrite and 43%–45% austenite. By further layer deposition, thermal cycle overlapping occurs and the area from the welded zone is reheated and thus favours the initiation of secondary phases precipitation phenomena. Both phenomena are specific to both the weld and the heat affected zone. To avoid these problems, it is necessary to focus the welding procedures to a minimum, maintaining the time at the highest reached temperature and to limit the linear energy and temperature between two successive passes. In a simplified way, it can be said that it is necessary to have an upper limitation of the welding heat input and of the temperature between the consecutive rows to prevent the formation of sigma phase, and an inferior limitation to avoid nitrides formation.

Regardless of the linear energy value used for the present experiments, the microstructure of the final layer for achievement the welded joint filling is biphasic and consists of 32%–34% ferrite and 66%–68% austenite (Figure 7).

**Figure 9:**
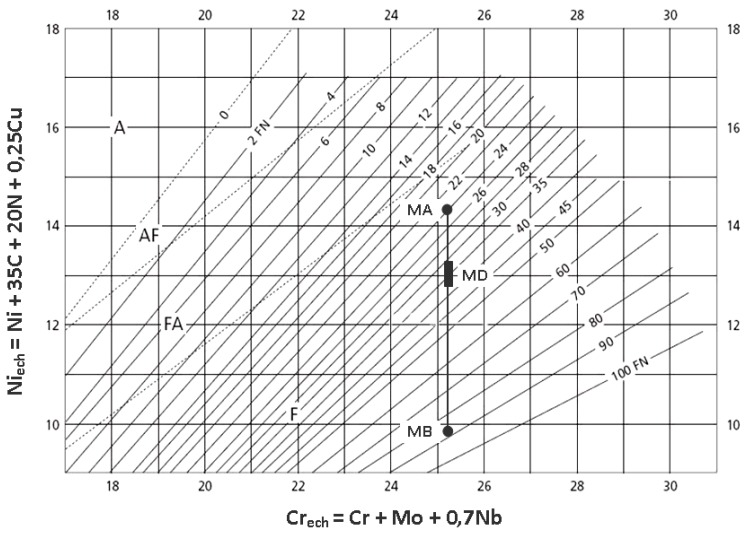
Ferrite diagram index with construction for ferrite index calculation.

The cooling time parameter between 1200 and 800 °C marked t_12/8_ provides a reference point for studying the welded joints microstructure [8,11,24]. Although its value increases as the welding linear energy grows, there were no differences in the relative amounts of ferrite and austenite. The reason for this is probably a consequence of the small measure variation of the cooling rate for the three values of the welding linear energy and of the high influence on the structure of the alloying elements that promote ferrite (Cr, Mo) and austenite (Ni, N). Normally, the ferrite content of the deposited metal must correspond to the ferrite index FN 30–100 (22%–70%) [8,17,18]. Transposing on the diagram WRC-92 [25] from Figure 9, the values Cr_ech_ and Ni_ech._ Specific to the base metal and filler material and taking into account the dilution degree value, the ferrite index of deposited metal was theoretically estimated to be FN 32–38 (24%–28%).

In the area from the heat affected zone (HAZ) adjacent to the fusion line, a predominantly ferritic structure occurs (approximately 58%–60% ferrite, Figure 8). The width of this zone is very narrow, 120–160 μm, so that it will not decrease the mechanical properties of the welded joint. The austenite nucleation took place on ferrite grain boundaries and inside them, with the appearance of the Widmanstätten structure (Figure 8b) and, because of the epitaxial growth of the crystalline grains, a columnar structure was formed (Figure 8a).

The limitation of the welding linear energy at the established optimal values, along with additional nitrogen alloying, reduces the grain size growth and σ phase precipitation tendency. In addition, there is a fine particle precipitation (Figure 8b) of plate-like Cr_2_N nitrides and M_23_C_6_ carbides [6]. As the linear energy is increased, the amount of ferrite in the weld and HAZ is lower and the risk of formation of intermetallic phases is higher. Moreover, the nitrogen presence as an alloying element inhibits the crystalline grain size growth, and due to its high solubility in the austenite, the proportion of the precipitated nitrides would not be very high. A structure with a high ferrite proportion may reduce the toughness at low temperatures, while a structure with a too high amount of austenite affects both the mechanical strength characteristics and stress corrosion resistance in chloride environments [13]. The sensitivity to hot cracking is reduced for very high concentrations of Ni and N. At the same time, the cold cracking resistance of the austenitic-ferrite welded joint and of the high ferritized zone from HAZ is high, although in austenitic areas, neighboured to the ferrite zones, a substantial amount of hydrogen can be stored. To eliminate the cold cracking risk, the selection of welding materials with low hydrogen content is recommended together with the application of preheating at about 150 °C [4,9,11].

### 3.3. EDX Analysis

During the molten metal bath solidification, the cooling rates, specific to the electric arc welding, are so high that they cause segregation of alloying elements. In addition, the transformation of ferrite in austenite is associated with the Cr, Ni, Mo and N redistribution between the two phases (Table 2).

These data demonstrate that the ferrite is enriched in Cr and Mo and becomes poor in Ni and N. As a result, the chemical composition of the ferrite is more alloyed with Cr and Mo compared to the average composition of the weld and of the austenite.

**Figure 10:**
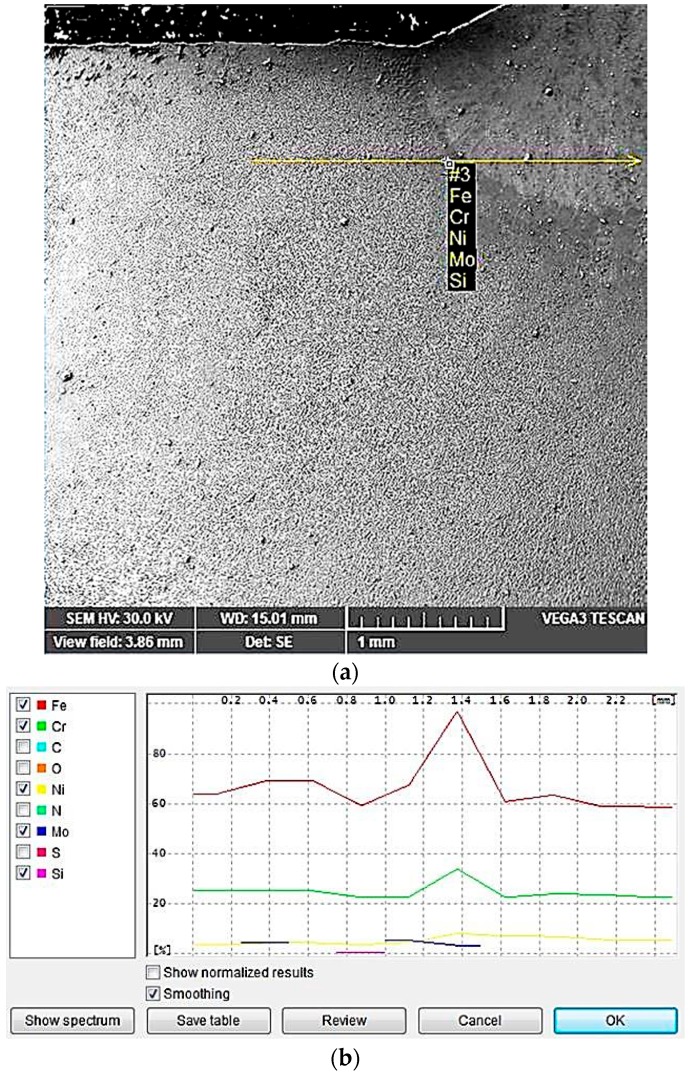
Examination of the last layer deposition with E_l_ = 15 kJ/cm: (**a**) SEM image; and (**b**) line scans showing the compositional variation for the region indicated in (**a**).

**Figure 11:**
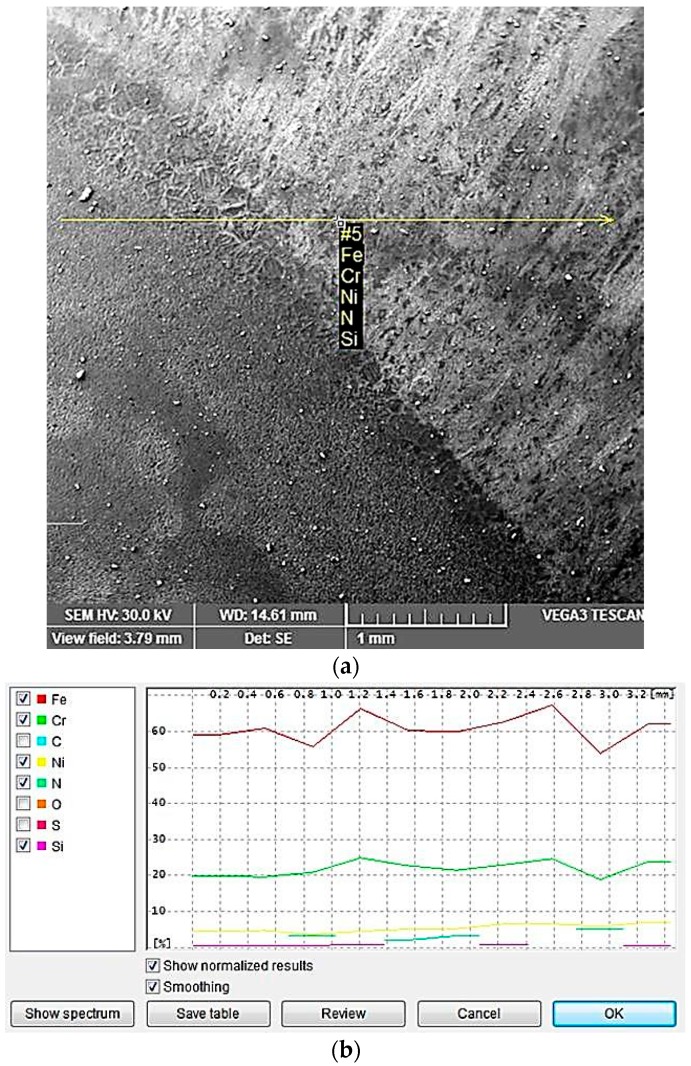
Examination of the median zone in the deposited layer with E_l_ = 15 kJ/cm; and (**a**) SEM image; (**b**) line scans showing the compositional variation for the region indicated in (**a**).

In Table 3 and Figure 10 and Figure 11, the concentration variation of Cr, Ni, Mo is shown in different parts of the welded joints, which have been realized with a linear energy E_l_ of 6.9 kJ/cm for the welded joint and of 15 kJ/cm for the filling layers.

Such variations in the concentration of the alloying elements in the weld and in the heat affected zone (HAZ) are based on both transformation and precipitation reactions taken place, occurring in a wide temperature range, generally comprised between 1300 and ca. 300 °C. It is highlighted that the redistribution degree of the alloying elements strongly depends on the overall thermal cycle specific to the welding operation.

Due to the high temperatures reached in the vicinity of the welding zone, even though the heat-affected zone is predominantly ferritic at the maximum temperature reached at welding, during the subsequent cooling, another solid state transformation occurs, with austenite formation. As a result, a redistribution of the alloying elements appears, comparable to that observed in welding.

### 3.4. X-ray Diffraction Analysis

Investigation by X-ray diffraction of the studied samples was performed using a Philips diffractometer (PAN alytical XPert Pro Multi-Purpose Diffractometer, Kassel—Waldau, Germany) equipped with a graphite monochromator for Cu-Kα radiation (λ = 1.54 A) at room temperature.

The measurements were carried out in 2 theta geometry, in the range of 20°–100° degrees, at a speed of 1°/min. It was operated at a voltage of 40 kV with a current intensity of 30 mA.

Crystallographic phases identification of the samples was performed using the database JCPDS (Joint Committee on Powder Diffraction Standards).

**Figure 12:**
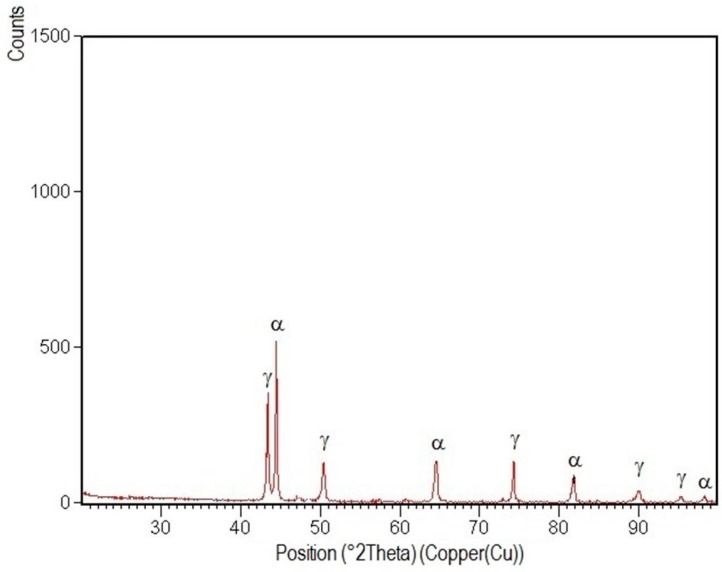
XRD pattern of the welded joint root layer realized with E_l_ = 6.9 kJ/cm.

**Figure 13:**
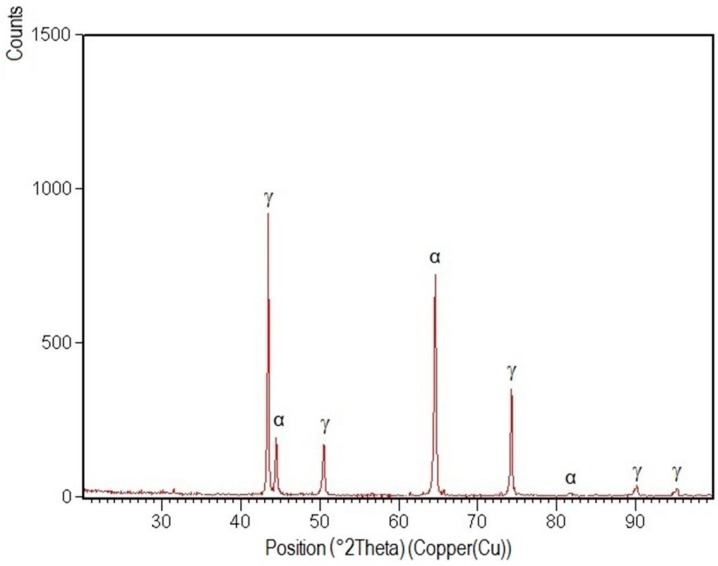
XRD pattern of the welded joint central zone E_l_ = 15 kJ/cm.

**Figure 14:**
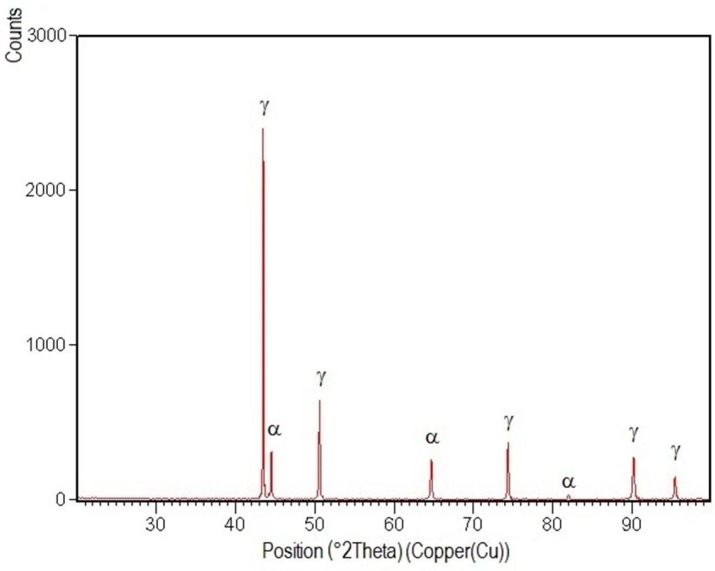
XRD pattern of the welded joint filling layer realized with E_l_ = 15 kJ/cm.

**Figure 15:**
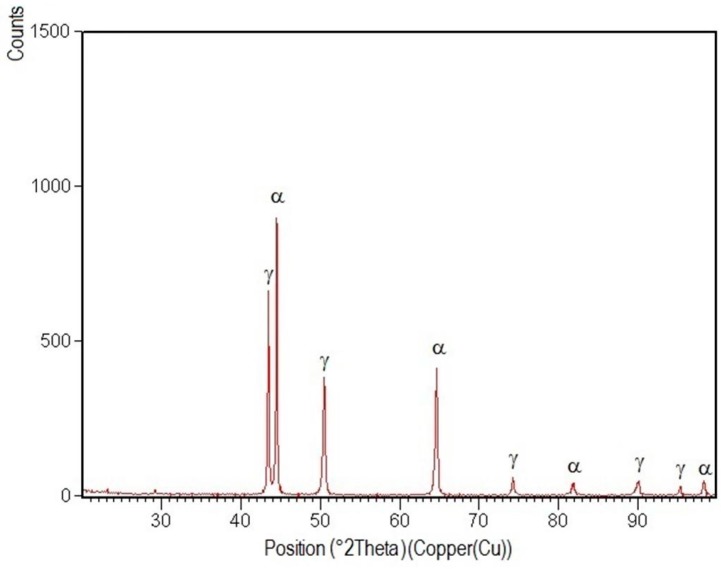
XRD pattern of the interface weld-HAZ for E_l_ = 15 kJ/cm.

**Figure 16:**
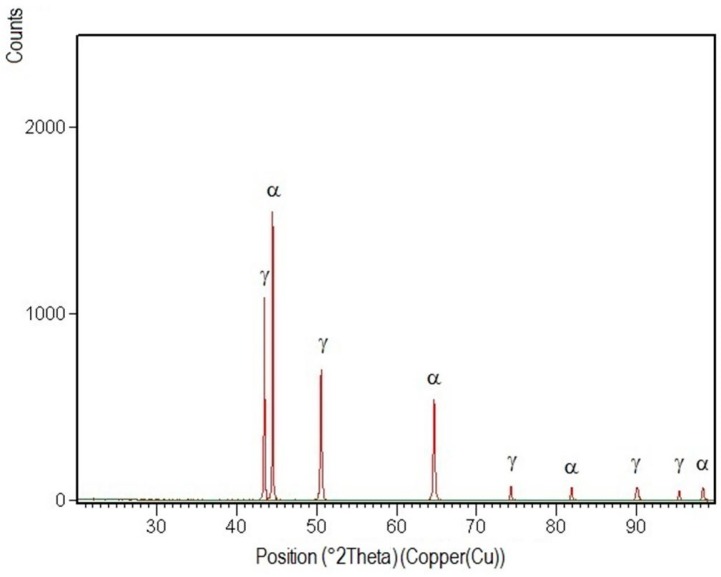
XRD pattern of the base metal.

XRD patterns specific to the welded joint areas are shown in Figure 12, Figure 13, Figure 14, Figure 15 and Figure 16.

After their indexation, the following results were obtained:

- Structural phases detected in all areas of the welded joints are ferrite and austenite.
- The high temperatures reached in the vicinity of the fusion line promote a predominantly ferrite microstructure in the HAZ zone (interference peaks are more pronounced), with a smaller proportion of austenite resulted from the solid state transformation, initiated in the welded joint cooling stage.
- The specific global thermal cycles during successive welding passes, beside the higher Ni content of the selected filler material compared to the base metal, justify the data obtained regarding the quantitative report of the two main phases (A,F) present in the final microstructure following primary and secondary crystallization.
- Both in the weld and HAZ zones, the intermetallic phases, which affect the mechanical properties of welded joints, were not highlighted.

## 4. Conclusions

1. Variation of the linear energy within the range values of 10 kJ/cm and 20.7 kJ/cm lead to welded joints with a suitable macro-geometry, without metal continuity defects such as porosity, cracks or slag inclusions.
2. The micrographic and X-ray diffraction analysis showed that for the welding conditions used, the base metal consists of approximately 51% ferrite and 49% austenite, and in the weld seam, the ferrite content decreases from the surface to the root layer.
3. The high cooling rates during primary and secondary crystallisation of the molten metal bath is associated with the segregation of the alloying elements and their redistributing between ferrite and austenite.
4. As a result of the high temperatures reached in the vicinity of the fusion line, with a small thickness of approximately 120–160 μm, its microstructure became predominantly ferritic and by subsequent cooling its partial transformation in austenite occurred.
5. The deposition of the root layer, with a linear energy of 6.9 kJ/cm, and of the filling layers, with values of 10 kJ/cm to 20 kJ/cm, prevents the cracking by liquation phenomenon of the weld and limits the fragile intermetallic phases’ precipitation in the welded joint area.

## Figures and Tables

**Table 1:** The chemical composition of Duplex X2CrNiMoN22-5-3 stainless steel, wt %.

C, %	Si, %	Mn,%	P, %	S, %	Cr, %	Ni, %	Mo, %	N, %
0.021	0.79	0.82	0.019	0.021	22.34	5.61	3.1	0.14

**Table 2:** Variation of the alloying elements concentration from the weld, for a linear energy, E_l_ = 15 kJ/cm.

Phase	Cr, wt %	Ni, wt %	Mo, wt %	N, wt %
Ferrite/Ferrite	21.61–23.83	5.40–6.22	2.62–3.35	<0.052
Austenite/Austenite	19.92–21.5	6.31–8.70	2.51–2.98	0.31–0.58

**Table 3:** EDX investigations on the welded joint zones.

Welded Joint Zone	Phase	Cr, wt %	Ni, wt %	Mo, wt %
Root weld	F/F A/A	23.62 23.14	7.16 7.85	2.98 2.67
Heat affected zone (ZIT)	F/F A/A	21.88 20.76	5.17 5.83	3.39 2.87
Weld-median zone	F/F A/A	24.84 22.60	6.18 8.55	3.68 2.47
Weld-outer zone	F/F A/A	23.71 23.42	7.20 7.72	2.73 2.54
Base metal	F/F A/A	23.18 19.47	4.08 7.16	3.29 2.43
